# Soybean-VCF2Genomes: a database to identify the closest accession in soybean germplasm collection

**DOI:** 10.1186/s12859-019-2859-5

**Published:** 2019-07-24

**Authors:** Jungmin Ha, Ho Hwi Jeon, Dong U. Woo, Yejin Lee, Halim Park, Joohyeong Lee, Yang Jae Kang

**Affiliations:** 1 Department of Plant Science and Research Institute of Agriculture and Life Sciences, Seoul National University, Seoul, Republic of Korea; 2 Plant Genomics and Breeding Institute, Seoul National University, Seoul, Republic of Korea; 3 Division of Applied Life Science Department at Gyeongsang National University, PMBBRC, Jinju, Republic of Korea; 4 Division of Life Science Department at Gyeongsang National University, Jinju, Republic of Korea; 5 College of Pharmacy and Research Institute of Pharmaceutical Sciences, Gyeongsang National University, Jinju, South Korea

**Keywords:** Soybean, Germplasm identification, Genome-based breeding, Next generation sequencing, Machine learning

## Abstract

**Background:**

The development of next generation sequencer (NGS) and the analytical methods allowed the researchers to profile their samples more precisely and easier than before. Especially for agriculture, the certification of the genomic background of their plant materials would be important for the reliability of seed market and stable yield as well as for quarantine procedure. However, the analysis of NGS data is still difficult for non-computational researchers or breeders to verify their samples because majority of current softwares for NGS analysis require users to access unfamiliar Linux environment.

**Main body:**

Here, we developed a web-application, “Soybean-VCF2Genomes”, http://pgl.gnu.ac.kr/soy_vcf2genome/ to map single sample variant call format (VCF) file against known soybean germplasm collection for identification of the closest soybean accession. Based on principal component analysis (PCA), we simplified genotype matrix for lowering computational burden while maintaining accurate clustering. With our web-application, users can simply upload single sample VCF file created by more than 10x resequencing strategy to find the closest samples along with linkage dendrogram of the reference genotype matrix.

**Conclusion:**

The information of the closest soybean cultivar will allow breeders to estimate relative germplasmic position of their query sample to determine soybean breeding strategies. Moreover, our VCF2Genomes scheme can be extended to other plant species where the whole genome sequences of core collection are publicly available.

**Electronic supplementary material:**

The online version of this article (10.1186/s12859-019-2859-5) contains supplementary material, which is available to authorized users.

## Background

Recent NGS technologies have enabled the draft genome assemblies for majority of commercially important crops. Moreover, third generation long read sequencing technology can refine the published draft assemblies into nearly complete genome by spanning gaps for further genomic analyses [1, 2] . As the reference genome sequences of several model plants and crops have been publicly available, re-sequencing approaches have been activated covering several thousand genome projects for *Arabidopsis thaliana* [3], rice [4] and etc.. For soybean, 302 wild and cultivated accessions were resequenced in ~11x depth to reveal domestication traces in genome [5]. Moreover SoySNP50K Illumina iSelect BeadChip were developed based on the reference genome sequence for intensive genotyping, and total 18,480 domesticated soybean and 1168 wild soybean accessions of Soybean Germplasm Collection was genotyped [6]. Also, Axiom SoyaSNP array was developed based on resequencing data of 31 soybean accessions covering larger portion of soybean genome than SoySNP50K for germplasm genotyping with higher resolution [7]. The Axiom SoyaSNParray and SoySNP50K is publicly available for further genomics researches.

Although the large genotype datasets are publicly downloadable, majority of non-computational researchers failed to utilize the large data into practical field due to the lack of easy-to-use tools. Most practical bioinformatic tools are available for command line console in Linux environment with high computational powers, while several web-based tools are currently providing basic analytic services such as network visualization, gene set profiling, data visualization etc. rather than practical needs [8]. As we are getting close to $100 genome era; lowering sequencing prices and analytic burdens, the practical needs for the precise identification of samples in biology and agricultural fields are now emerging rather than rough phenotypic inspection. Especially, this discussion has been established for plant diseases to develop a NGS-based pathogen identification framework [9]. Also, the web server for inferring phylogenetic tree for microorganisms based on resequencing approach was published [10]. In agriculture genomic homogeneity of commercial seeds need to be ensured for stable yield in each year, and reliable certification of elite cultivars that have chosen by farmers is also necessary. Considering the impact of cultivars on various planting strategies or locally prevalent diseases in agriculture, verifying cultivars using resequencing technology would be critical to secure yield [11, 12].

The precise identification of unknown/query sample to a known cultivar requires rich information of genetic markers that would show distinct pattern for each cultivar. For the identification of soybean cultivar, researchers recently developed barcoding system that implements PCR for ~ 200 InDel markers converting them into two dimensional barcode for easy detection [13]. However, cheap sequencing prices along with advance in sample pooling technology eventually lowered the cost of resequencing, which makes, consequently, PCR practices more expensive than NGS, considering the cost of working time and labors. After processing the general workflow from quick sample preparation to sending the samples to sequencing facility for the resequencing, users would be provided with compressed raw fastq files, mapping files in BAM format and variant call files in VCF format. To identify the genomic background of unknown samples, a researcher needs to build genotype array and patch it with pre-built genotype matrix of diverse sample collection for phylogenetic tree construction. Several utilities have been developed to deal with VCF files such as vcf-kit; a software for easy handling of VCF format files [14]. However, these are mainly designed for bioinformatic researchers rather than non-computational users. Here, we developed easy-to-use web application which identifies the closest soybean accession of an unknown/query sample by whole genome sequencing result; VCF file. For alleviating computational burden, we abstracted the genotype matrix of soybean genotype collection using principal component analysis (PCA).

As whole genome sequences of global soybean germplasms have been being accumulated in public databases, the precise identification of the closest soybean accession may allow us to estimate phenotype based on the known phenotype information of the identified soybean accession in the database. Moreover, the accumulated large genotype dataset would be fundamental stepping stone for phenotype prediction based on intensive phenotyping effort [15]. Using our web-application “Soybean-VCF2Genomes”, the closest accessions to unknown sample can be easily identified on the web browser and it would help researchers or breeders to understand their plant materials precisely.

## Construction and content

### Reference genotype matrix construction

For construction of reference genotype matrix, we utilized previously published soybean 222 validation germplasm consisting of 83 cultivars (*Glycine max*), 79 wild accessions (*Glycine soja*), and 66 breeding lines including F2 and RIL with some redundancy [7]. This dataset consists of soybean germplasm from Korea, China and Japan, and few western accessions and it would be advantageous for construction of reference genotype matrix to cover the allele space as large as possible because the North-eastern Asia counties are presumably considered as the origin of soybean domestication [16]. Furthermore, the dataset is originally designed to develop Axiom SoyaSNP array with known clusters such as wild/cultivating soybean, landraces/breeding population, and some redundant accessions for validating SoyaSNP array-based genotyping. These known clusters are also beneficial to validate our clustering result.

First of all, we filtered out the SNP positions with minor allele frequency (MAF). We selected a strong criteria, 0.2, to leave only common alleles that would provide strong signal for clustering and filtered out the SNP positions with MAF lower than the criteria. After filtering out the SNP positions, we determined 61,875 SNP positions of 222 accessions (Additional file 1). Diploid genotypes of filtered SNP positions were scored and transformed into 20 principal components (PC) with sum of explained variance ratio, 0.52, using sklearn package to build reference genotype matrix [17]. It resulted in 20 × 222 reference genotype matrix.

**Fig. 1:**
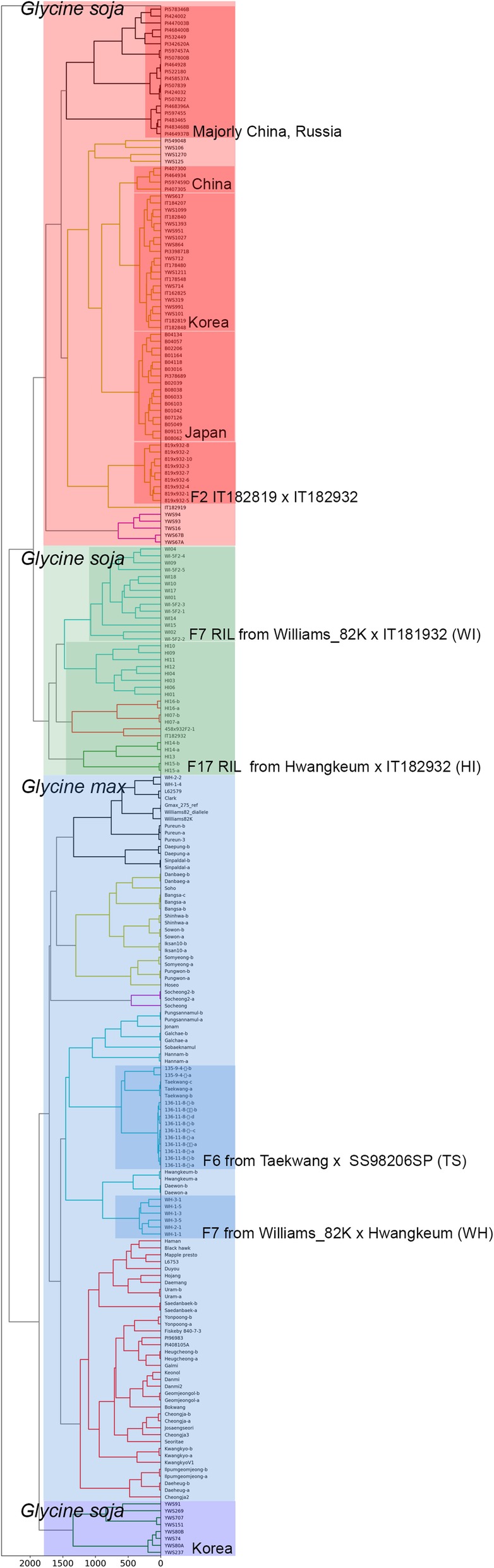
Phylogenetic tree constructed by genotype matrix. Genotypic data were transformed by PCA to save computing power. The classification agrees well with known origin of germpalsm

This light weighted genotype matrix could highly reduce computational burden of hosting server for hierarchical clustering trading off the information for clustering. However, the dendrogram based on clustering with the reference genotype matrix showed consistent phylogenetic relationship with known clusters such as wild/cultivated soybean, landrace/breeding populations, and redundantly assigned accessions (Fig. 1). Most importantly, the breeding populations from artificial crossing such as WI, HI, TS, and WH were well clustered each other. This result validate the PCA-transformed reference genotype matrix can cluster the soybean accessions precisely.

### Identification of similar accessions to query VCF file

To test whether the PCA-transformed reference genotype matrix can retrieve similar accession to query VCF files, additional VCF files were prepared from the publicly accessible NGS reads with more than 10X coverage in the National Center for Biotechnology Information (NCBI) sequence read archive (SRA), and the National Agricultural Biotechnology Information Center (NABIC) in Korea (Additional file 2). Read alignments were implemented with software BWA [18] followed by samtools, bcftools [19], and sambamba [20] as following.

# read mapping

> bwa mem Gmax_275_v2.0.fa sample_1.fastq sample_2.fastq -o out.sam

> samtools fixmate -O bam out.sam out.bam

> sambamba sort -o out.sort.bam out.bam

# SNP calling

> bcftools mpileup -Ob -o out.bcf -f Gmax_275_v2.0.fa out.sort.bam # or bamfiles

> bcftools call -vmO z -o out.vcf.gz out.bcf

The single VCF files were converted into numeric scores by pre-trained PCA parameters and clustered with the reference genotype matrix. The pre-trained PCA parameter was calculated when we construct the reference genotype matrix. We will re-calculate the PCA parameter when we update the reference genotype matrix. We implemented hierarchical clustering with method, ‘complete’, by Scipy package [21]. Resulting clusters were visualized into dendrogram with python matplotlib package [22]. The resulting panel displays the hierarchical clustering dendrogram and genotype distance values within sample cluster group.

**Fig. 2:**
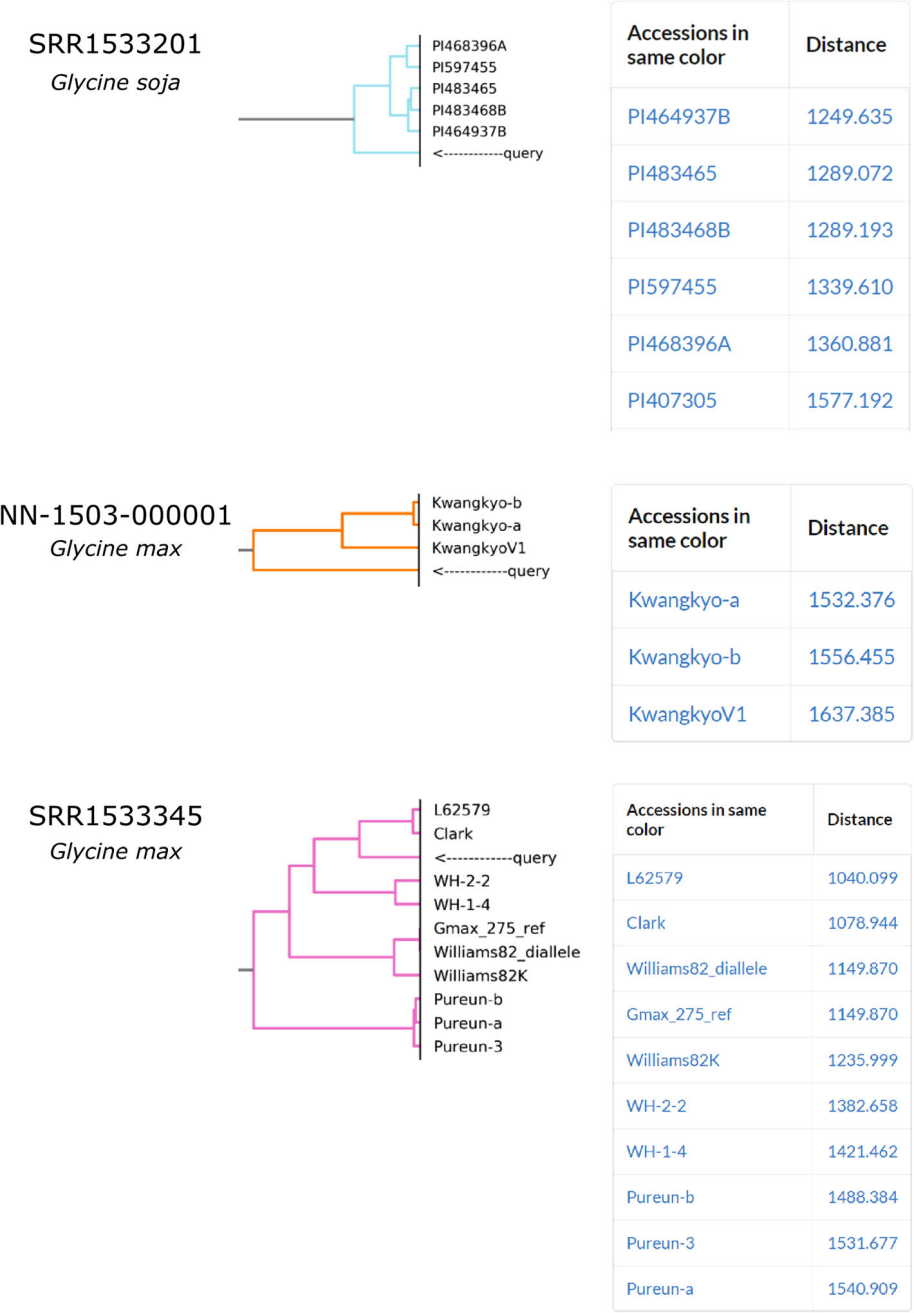
Clustering with single VCF file. A soybean cultivar Baegun was mapped to its known parental line; a *G. max* and G.soja accessions were properly mapped with *G. max* and *G. soja* clusteres, respectively

*Glycine soja* accession (SRR1533201, Alias1: IGDB-TZX-7991, Alias2: ZJ-Y282, Origin: Zhejiang, China) was mapped to wild soybean cluster consisting of the accessions from China [23]. The closest accession was PI464937B of which the origin is Jiangsu in China. *Glycine max* accession (SRR1533345, Alias1: IGDB-TZX-012, Alias2: PI548379, Origin: Heilongjiang, China) was mapped near soybean cultivar Clark. Its breeding pedigree is started from the cultivar Mandarin (PI548379) whose origin is also Heilongjiang, China. Another *G. max* accession (NN-1503-000001, NABIC) known as cultivar, “Baegun”, was mapped to soybean cultivar cluster and the closest cultivar was “Kwangkyo”, which is known as one of parents of “Baegun” [24]. These results suggest that our approach is working properly and can estimate parental lines if they are already in reference genotype matrix (Fig. 2).

### Continuous improvement of reference genotype matrix by accumulating public resequencing data

**Fig. 3:**
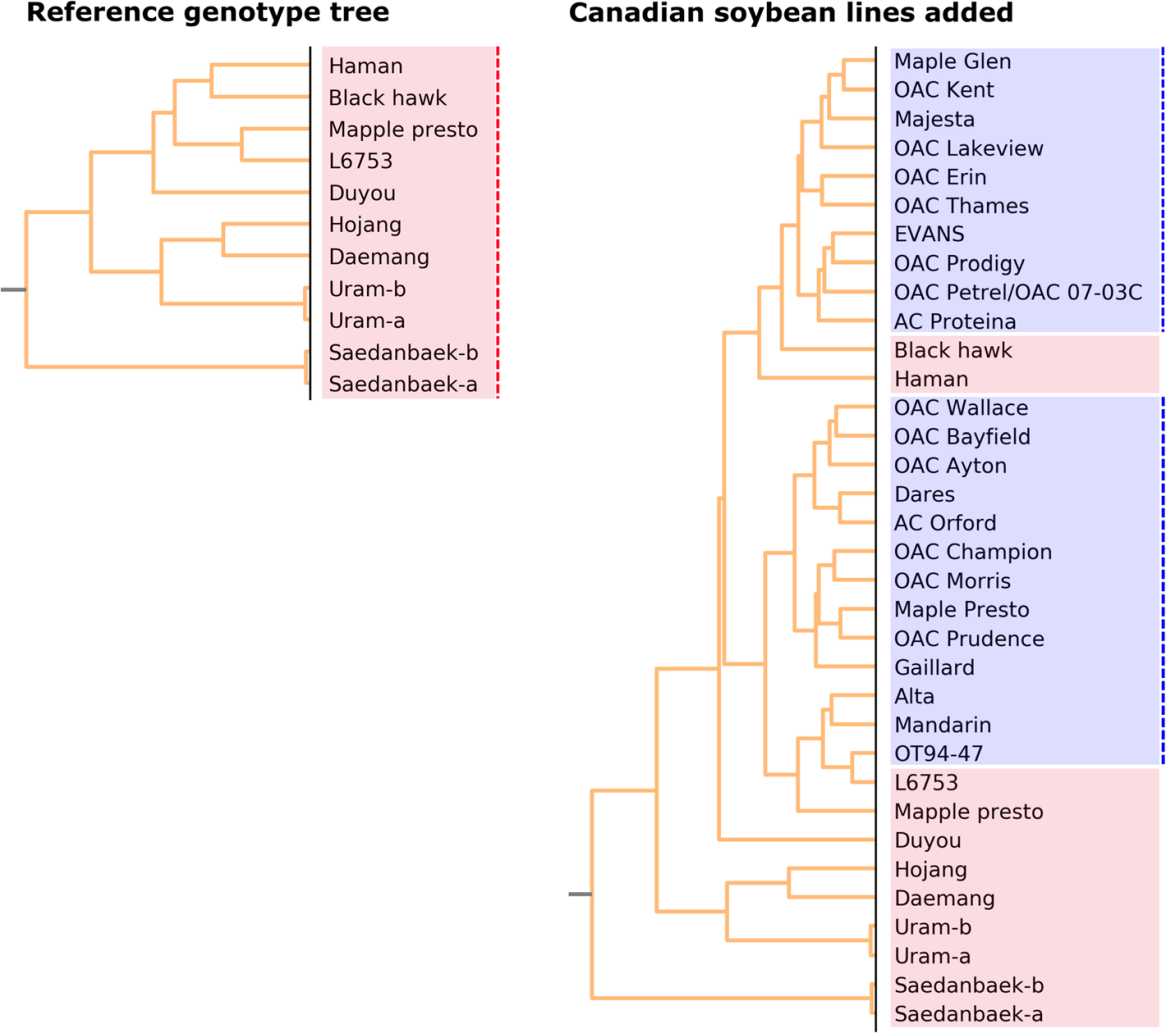
Integration of reference genotype matrix with additional accessions in NCBI. Genotypes of the Canadian soybean lines in study ID SRP059747 in NCBI SRA were integrated in our PCA-transformed reference genotype matrix. Red shaded accessions were included reference genotype matrix and blue shaded accessions are newly integrated Canadian soybean cultivars

With increasing number of soybean resequencing projects, it would be practical if we can integrate multiple sequencing deposits from public DBs into our reference genotype matrix. To test the applicability of pre-trained PCA parameters to additional *Glycine max* set that are not included in our reference genotype matrix (eg. Western soybean germplasm), we downloaded Canadian soybean accessions that are publicly available in NCBI SRA (Additional file 2) [25]. After SNP calling of the Canadian cultivars, we transformed the genotypes into numeric scores using pre-trained PCA parameters and integrate them with our reference genotype matrix. The resulting tree showed that the Canadian accessions are mapped near the “Mapple Presto” that has been maintained at Rural Development Administration in Korea with slight different name to “Maple Presto” in the Canadian accessions (Fig. 3). This shows that additional soybean accessions from other projects can be easily integrated into the reference genotype matrix for continuous update.

### Web interface of soybean-VCF2Genomes

**Fig. 4:**
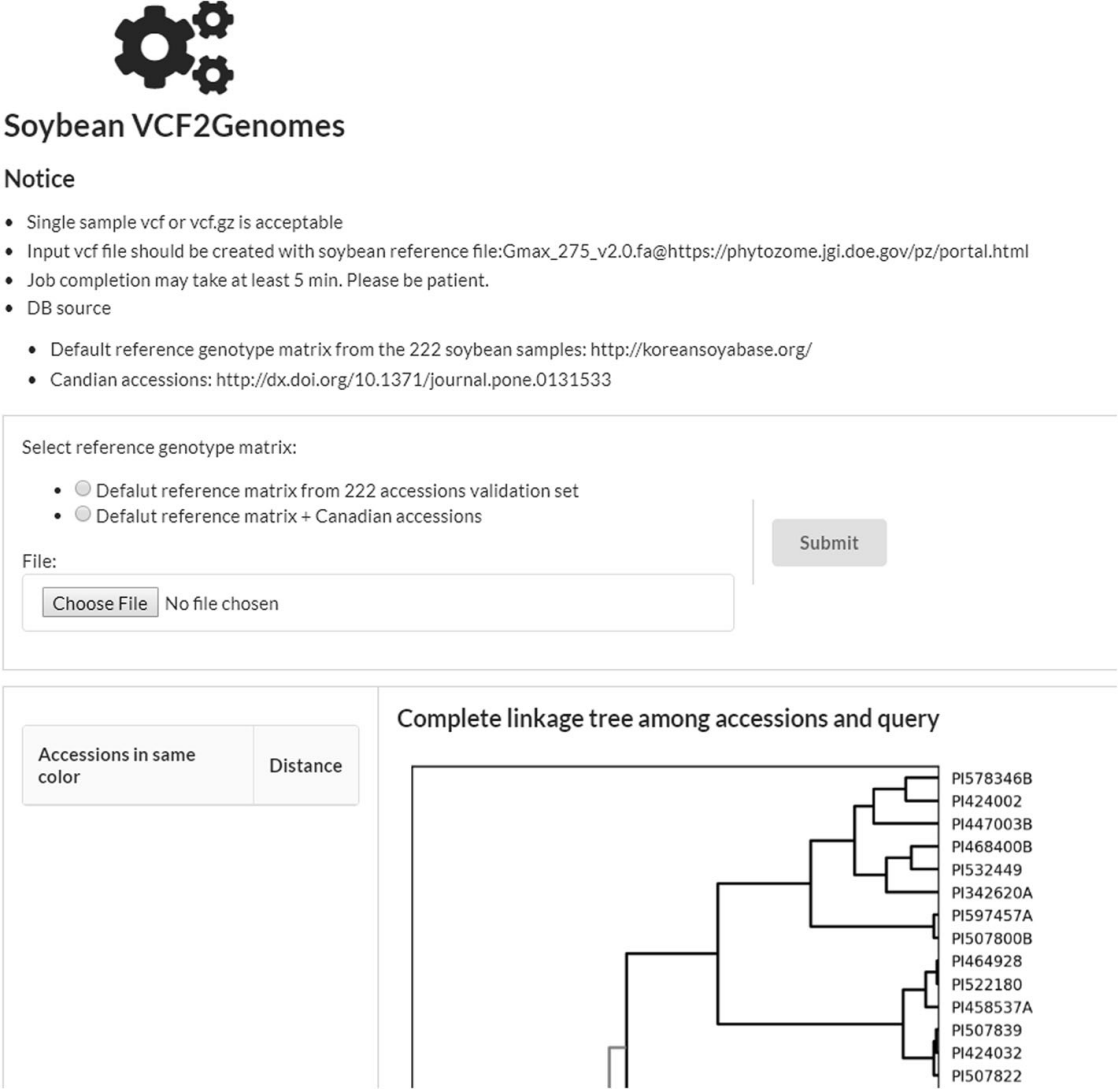
Web interface of Soybean-VCF2Genomes

We built our web application, Soybean-VCF2Genomes, using Django (Version 1.5, https://djangoproject.com) and semantic UI (https://semantic-ui.com/) (Fig. 4). Web interface is simply designed for user to choose the reference genotype matrix that currently two matrix are available; default 222 sample matrix and default + Canadian sample matrix. User can upload their single sample VCF file via file upload form. The result panel will display the image of hierarchical clustering tree in high resolution with query position as well as the distance value table. Total analysis time would be ~ 5 min after completion of VCF file upload.

### Utility and discussion

Soybean-VCF2Genomes identifies similar soybean accessions in reference genotype matrix for input VCF file with more than 10X genome coverage. The reference genotype matrix would be updated with the public NGS data of representative soybean germplasm. Currently ~ 1800 soybean accessions are available in NCBI SRA and more and more accessions are expected to be deposited in near future. However, the meta information of the deposited data is not always in ideal format and it is necessary to curate the labels (eg. name of cultivar, germplasm ID) of the accessions and select representative accessions according to the geographical origin and known pedigrees to improve our reference genotype matrix. The efforts for continuous update will eventually allow label identification of query sample rather than similar accession retrieval.

The identification relative phylogenetic position among the available soybean germplasm space would be beneficial to estimate rough phenotype of unknown samples based on the known phenotype information of the closest accession in reference genotype matrix. This would be also quick solution when researchers are trying to determine the rough origin of the wild or landrace accessions collected from local regions for many purposes such as genetic resource collection and import/export quarantine. Also, this will be useful for validation of commercial cultivars to prove its genomic background. Moreover, breeders can estimate relative distance of their crossbreeding combinations based on the reference genotype matrix to determine the plan of annual field trial. Our web application accepts directly VCF file which the sequencing service providers usually returns to customers. Hence, it is easy-to-use without any bioinformatic knowledge.

One limitation is that Soybean-VCF2Genomes currently supports only single sample VCF file due to the hosting burden and file transfer issues. As it contains only variant information, the compressed single sample VCF file size varies from 70Mbytes to 150Mbytes. However, the multi-sample VCF files (> 10 samples) would be too large to transfer as it reaches several hundreds or thousands Mbytes. Supporting multi-sample VCF file would be possible if we can host Soybean-VCF2Genomes at cloud server that support fast file transfers and flexible storage and memory.

## Conclusion

As whole genome sequences of global soybean germplasms have been accumulated in public databases, the identification of genomic background of unknown/query samples would be possible using reference genotype matrix. Here, we built light weight reference genotype matrix using PCA and web-application, Soybean-VCF2Genomes, where user can upload their single sample VCF file for analysis. Using the web-application, the closest accessions to unknown/query sample can be easily identified and it would help researchers or breeders to understand their plant materials precisely.

## Additional files

Additional file 1: MAF filtered genotype matrix of soybean germplasm. (XLSX 50509 kb)

Additional file 2: NCBI SRA accessions for testing germplasm clustering. (XLSX 12 kb)

## Abbreviations

NABIC: National agricultural biotechnology information center
NCBI: National center for biotechnology information
NGS: Next generation sequencer
PCA: Principal component analysis
SRA: Sequence read archive
VCF: Variant call format
